# *Naegleria fowleri* outbreak in Pakistan: unveiling the crisis and path to recovery

**DOI:** 10.3389/fpubh.2023.1266400

**Published:** 2023-10-19

**Authors:** Abdullah Nadeem, Inshal Arshad Malik, Eesha Khan Afridi, Fariha Shariq

**Affiliations:** ^1^Department of Medicine, Dow University of Health Sciences, Karachi, Pakistan; ^2^Department of Medicine, Jinnah Sindh Medical University, Karachi, Sindh, Pakistan; ^3^Department of Medicine, Karachi Medical and Dental College, Karachi, Sindh, Pakistan

**Keywords:** *Naegleria fowleri*, primary amebic meningoencephalitis, outbreak, treatment options, public health, Pakistan

## Abstract

The outbreak of *Naegleria fowleri* in Pakistan presents a significant public health concern due to its high fatality rate and limited treatment options. This review explores the impact of the outbreak on communities and the challenges faced in combating the disease. It evaluates available treatment options and highlights the need for early diagnosis and intervention. The study proposes recommendations to improve public health preparedness, including public awareness campaigns, enhanced healthcare infrastructure, and robust water surveillance systems. Collaboration between research institutions and public health organizations is emphasized to develop effective outbreak response strategies.

## 1. Introduction

*Naegleria fowleri* known more frequently as the “brain-eating ameba,” is a free-living ameba species belonging to the *Naegleria* genus and it is the only pathogenic species of the genus (1). It is the causative agent of Primary amebic meningoencephalitis (PAM), an infection that is rare but has a mortality rate of 95–99% (2). It is a thermophilic microorganism that flourishes at elevated temperatures of up to 46°C (115°F) and may endure even greater temperatures for brief periods of time (1, 2). As such, it is found in warm freshwater bodies and soil. Apart from that, it may also be present in swimming pools, splash pads, surf parks, or other recreational venues that are inadequately maintained and insufficiently chlorinated. Its thermophilic nature also explains why it is more likely to cause infection in the summer season (1).

The ameba can enter the nasal cavity when swimming or diving in contaminated water bodies or as is more commonly seen in Pakistan, irrigating the nose with contaminated water as part of ritual ablution. From here, it makes its way into the brain and starts devouring the brain tissue thus deriving the name “brain-eating ameba” (3).

According to a study, Pakistan had the second highest prevalence of *Naegleria* infections around the world (4). The first case of PAM in Pakistan was recorded in Karachi in October 2008. Within a decade of this, the number of cases in Pakistan had overtaken those reported in the USA in a span of 50 years (5, 6). As of 2023, *Naegleria* has claimed seven lives in Pakistan. Of these, six deaths were reported in Karachi (four cases originating in Karachi, one in Hyderabad, and one in Quetta)^1^ and one in Lahore.^2^

It has been pointed out that while Pakistan produces < 1% of the world's greenhouse emissions, it is disproportionately vulnerable to the wide-ranging effects of climate change because of its geographic location (7). One of these effects has substantialized in the form of worsening heat waves (8) that provide suitable temperatures for *N. fowleri* which is already a growing problem for the country.

Additionally, recent mortalities have raised a question about the maintenance and chlorination of recreational water bodies and tap water provided by the Karachi Water & Sewerage Board (KWSB) in the city that may be housing *N. fowleri*^1^ (9). Moreover, the COVID-19 pandemic unveiled some significant deficiencies in Pakistan's healthcare system's ability to provide health equity (10) and the country simply cannot afford another outbreak, that too with such a high rate of mortality.

Thus, keeping all of this in mind, it is of vital importance that this infection is recognized and timely addressed to prevent further morbidity and mortality.

## 2. Understanding *Naegleria fowleri*

*N. fowleri*, belongs to the Percolozoa phylum (20) and is primarily transmitted through water, although another method of infection is through dirt or dust (11, 12).

As a free-living protist, *N. fowleri* mostly consumes bacteria, both Gram-positive and negative, along with yeast, algae, and other microorganisms. *N. fowleri* responds to bacteria by forming food cups, engaging in chemotaxis, and secreting chemokines (13, 14). While more than 40 species of *Naegleria* have been found, only *N. fowleri* causes primary amebic meningoencephalitis (PAM), a fatal brain infection (2, 15).

### 2.1. Habitat of *Naegleria fowleri*

This thermophilic microorganism can be categorized into two groups based on its habitat, with one group inhabiting natural settings and the other being found in urban areas. Natural habitats include locations like hot springs, warm water bodies, ponds, freshwater lakes, and rivers, while urban areas may harbor *N. fowleri* in drinking water distribution systems (DWDS) within pipe wall biofilms (16). It can also be encountered in various settings such as hospitals, geothermally heated water sources, contaminated drinking water supplies, water parks, dental unit waterlines (DUWLs), and instances where nasal exposure to tap water occurs, including swimming pools in hotels and homes. Moreover, the prevalence of *N. fowleri* tends to be higher in regions where the water temperature reaches or exceeds 28°C (17).

### 2.2. Life cycle of *Naegleria fowleri*

*N. fowleri*'s life cycle is divided into three phases: trophozoites, cysts, and flagellates (18).

**Figure 1:**
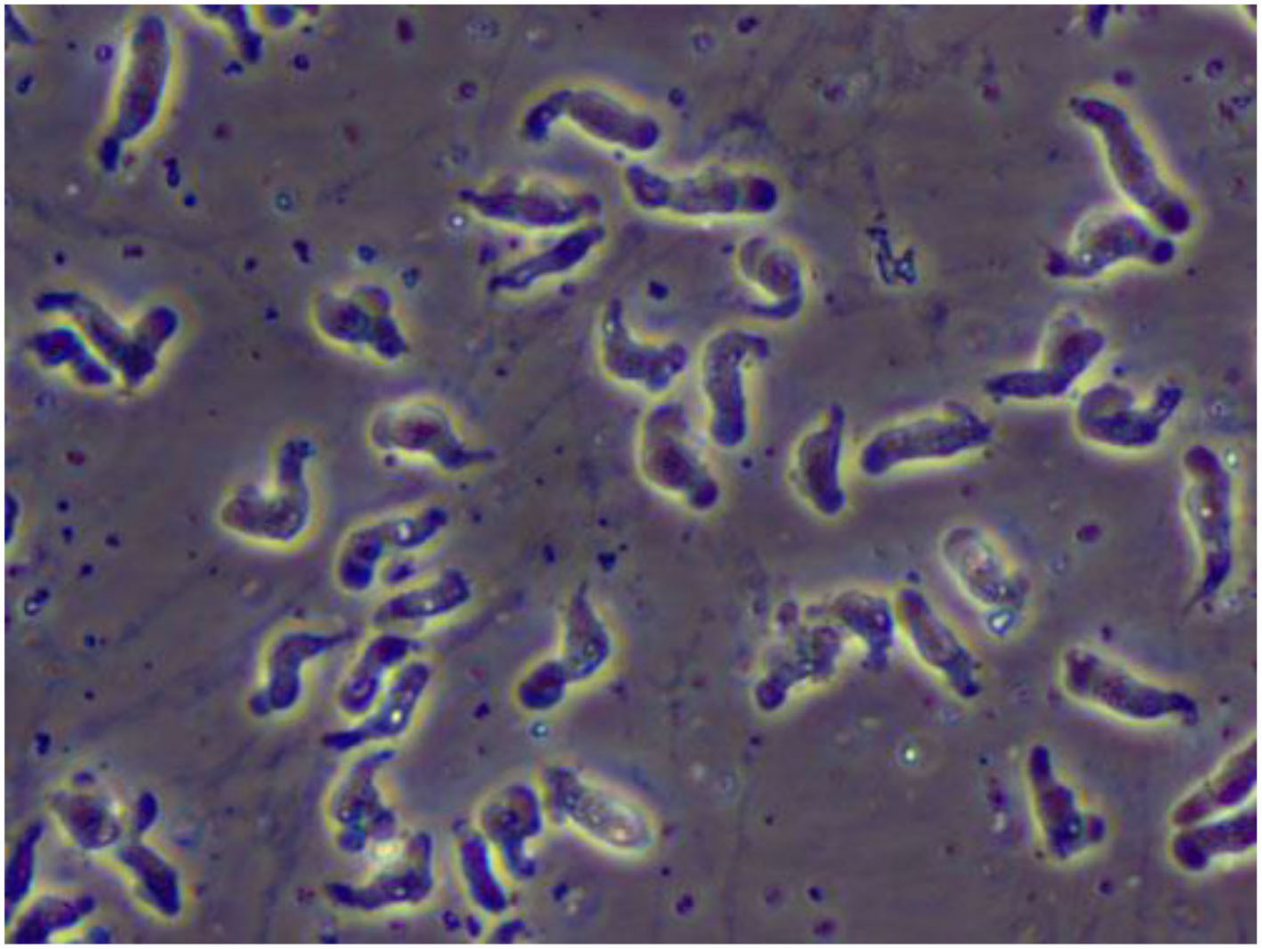
A wet mount of *Naegleria fowleri* trophozoites cultured from a patient of primary amebic encephalitis (PAM) (from https://www.cdc.gov/parasites/Naegleria/Naegleria-fowleri-media.html).

The ameboid trophozoite shown in Figure 1, is the active, reproducing and feeding stage of *N. fowleri* (19). These trophozoites enter the body through the nasal tissue and travel to the brain via the olfactory nerves, resulting in a condition known as primary amoebic meningoencephalitis (PAM). Once *N. fowleri* reaches the olfactory bulbs, it elicits a significant immune response through activation of the innate immune system, including macrophages and neutrophils. Among the three stages, only the trophozoite is capable of causing infection in humans (20). When faced with certain conditions, such as a scarcity of nutrients, trophozoites can temporarily transform into a non-feeding flagellated stage. They can then revert back to the trophozoite form when favorable conditions return (18). Trophozoites of *Naegleria fowleri* primarily localize in the tissues of the central nervous system, particularly within the brain. However, flagellated forms of the organism are typically observed in cerebrospinal fluid (CSF) when the CSF is intentionally diluted for diagnostic flagellation tests. These tests are conducted to evaluate the presence and characteristics of the flagellated form, aiding in the diagnosis of *N. fowleri* infections.

**Figure 2:**
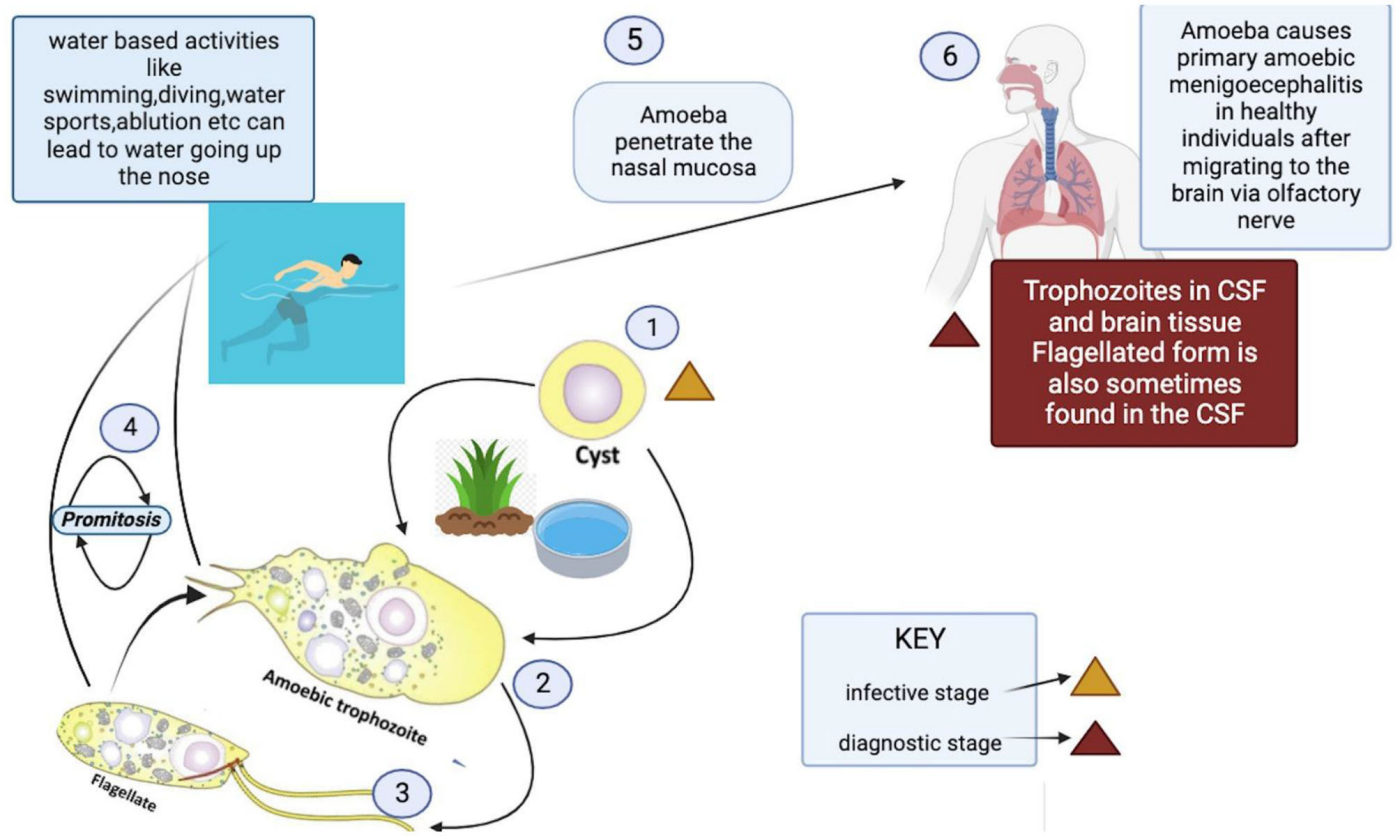
Life cycle and transmission route of *Naegleria fowleri*.^3^

The third form is a dormant spherical cyst. Notably, cysts are not detected in brain tissue (21). Trophozoites or flagellates encyst under unfavorable environmental circumstances such as lack of nutrients, overcrowding, desiccation, the buildup of waste materials, and extreme temperatures (22) (Figure 2). This enhances the chances of survival until better environmental conditions are present.

### 2.3. Transmission routes for *Naegleria fowleri*

PAM is substantially more common in immunologically healthy people, healthy children, and young adults who have recently been exposed to recreational freshwater (23). Since PAM is a waterborne illness, the majority of cases are linked to diving and swimming in under-chlorinated pools, polluted canals, spas, or engaging in recreational activities like water skiing in contaminated water sources, as well as the use of neti pots for nasal cleansing and ablution (24). When contaminated water is forced or splashed into the nasal cavity under pressure during swimming, diving, or otherwise, the ameba colonizes the nasal cavity. After nasal inoculation, the ameba passes through the respiratory epithelium and attaches to the olfactory mucosa. The pathogen then moves along the olfactory nerve and past the cribriform plate, which is more permeable in children and young adults, to reach the olfactory bulbs within the central nervous system (CNS) (11). Once *N. fowleri* enters the olfactory bulbs, it triggers the innate immune system, which includes neutrophils and macrophages, to produce a strong immunological response (12, 13).

## 3. Clinical manifestations and signs and symptoms

**Figure 3:**
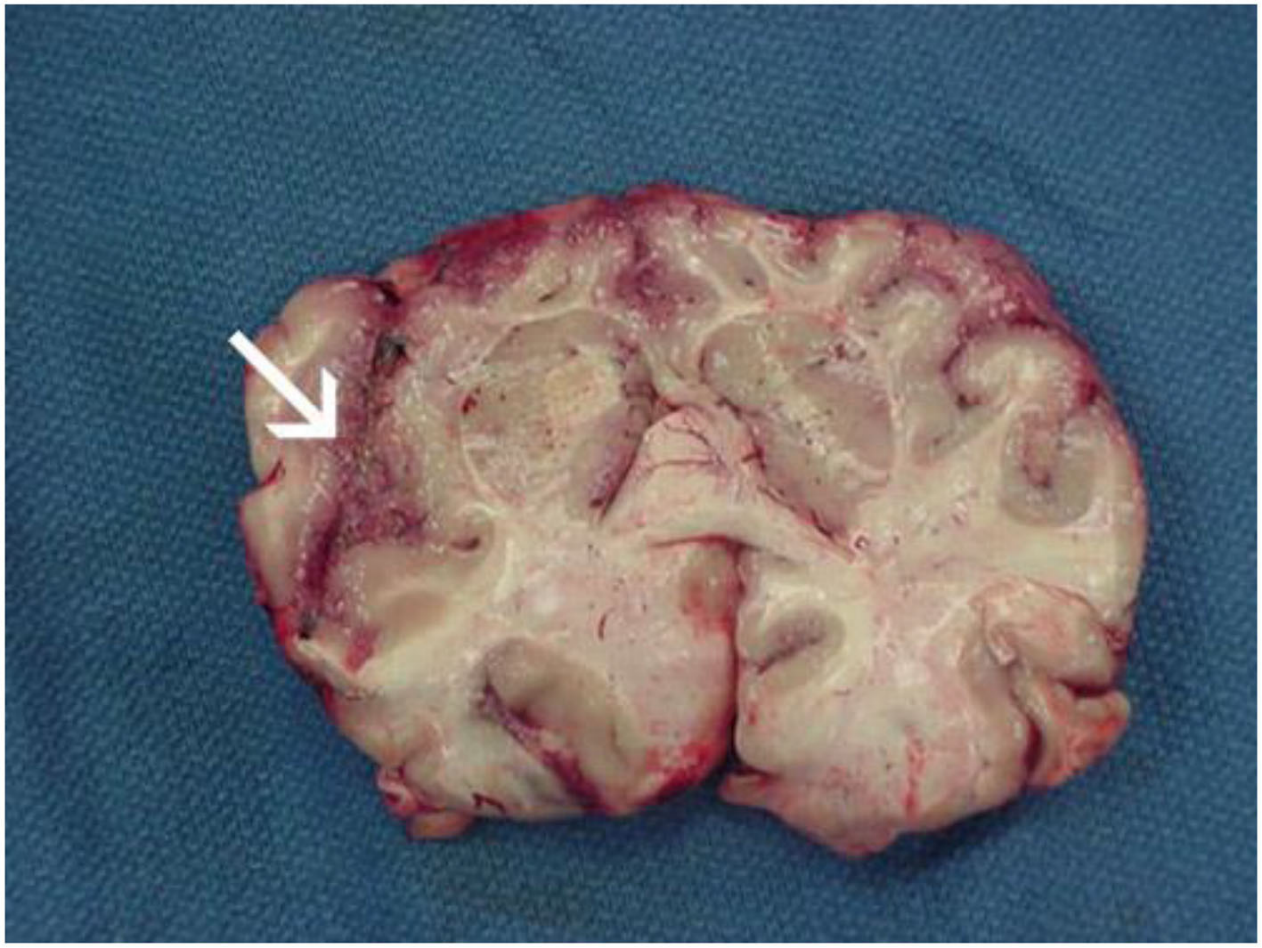
Focal hemorrhage and necrosis in frontal cortex due to *Naegleria fowleri* (from https://www.cdc.gov/parasites/Naegleria/Naegleria-fowleri-media.html).

PAM is characterized by symptoms that resemble those of bacterial or viral meningitis, such as fever, stiff neck, headache, vomiting, anorexia, and seizures (25). Fatigue, headaches, nausea, and vomiting are a few of the early signs of the illness. Later, more serious symptoms including confusion, neck stiffness, photophobia, seizures, and cranial nerve abnormalities start to appear (18) (Table 1). The amount of time between initial contact with the pathogenic *N. fowleri* strain and the onset of clinical symptoms can range from 2 to 3 days to as long as 7–15 days, depending on the strain's virulence and the size of the inoculums (27). Clinical signs like changes in smell perception and respiratory infections indicate involvement of the olfactory epithelium and invasion of brain tissue (28). Before showing signs of PAM, such as meningitis, patients with PAM do not exhibit any nasal irritation symptoms, such as pain, bleeding, tenderness at the bridge of the nose, sneezing, and/or prolonged rhinorrhea (25). Severe headache, fever, chills, positive Brudzinski and Kernig signs, photophobia, confusion, seizures, and potential coma are among the most typical symptoms. In a few cases, myocardial necrosis and aberrant heart rhythms have also been reported (29). The apparent correlation between elevated cerebral spinal fluid (CSF) and intracranial pressure and mortality is, arguably, the most significant finding. Patients with *N. fowleri* infections have been shown to have CSF pressures of 600 mm H2O (28). According to CSF analysis, there are several color anomalies, ranging from gray in the early stages of infection to red in the late stages of sickness because of a large increase in red blood cells (30, 31). Additional increases in trophozoites and polymorphonuclear cell densities (up to 26,000 mm^3^) are also observed (28, 29). In the majority of cases, primary amebic encephalitis advances quickly, resulting in hemorrhage, coma, and death (18) (Figure 3). Death usually occurs 3–7 days following the onset of these symptoms (25).

**Table 1:** Onset signs and symptoms of *Naegleria fowleri* infection (26).

**Onset**	**Signs and Symptoms**
Early	Fever Headache Nausea Vomiting Changes in smell and taste
Late	Stiff neck Fatigue Hemorrhage Confusion Changes in personality Hallucinations Seizures Coma

## 4. Virulence of *Naegleria fowleri*

*N. fowleri*'s pathogenicity can be attributed to two primary characteristics. First, *N. fowleri* may consume brain tissue by a sucker-like surface structure known as a “food cup”. These feeding cups are created by the phagocytic activity of *N. fowleri*, which is mediated by Nfa1 and Nfactin (32). Second, *N. fowleri* secretes a variety of cytolytic chemicals, such as neuraminidases, acid hydrolases, phospholipases, and phospholipolytic enzymes, which can damage nerves in the central nervous system (CNS). Additionally, *N. fowleri* infection triggers a strong immune response that further damages the CNS (33). Although not fully understood (32), it is believed that *N. fowleri* has also developed mechanisms that inhibit the host immune system (34).

## 5. Replication of *Naegleria fowleri*

*N. fowleri* reproduces through a process known as binary fission, a form of asexual reproduction that allows a single organism to divide and generate two identical offspring (2). Reproductive division in *N. fowleri* involves promitosis, which occurs without the breakdown of the nuclear envelope (35). During this process, the mature parent cell undergoes replication of its genetic material while simultaneously increasing in size. The DNA within the parent cell then migrates toward opposite poles, and eventually, the cell membrane undergoes division, resulting in the formation of two daughter cells that are genetically indistinguishable from each other and from the parent cell (2).

## 6. Epidemiology of *Naegleria fowleri*

*N. fowleri* may be found anywhere around the world except for Antarctica (36), but it is dominant in certain geographic areas that offer suitable conditions for its survival and growth. Statistics show that infections *with N. fowleri* have been documented in 39 different nations. However, Pakistan, Mexico, Australia, the Czech Republic, and India have been the countries most impacted, along with the United States of America (USA) (19). This is because *Naegleria* thrives in hot temperatures and may be more prevalent in certain regions of these countries with warmer temperatures and an abundance of warm freshwater bodies, such as lakes, ponds, and poorly maintained swimming pools. For instance, in the USA, they were more prevalent in the Southern states (37) that have hotter temperatures (38).

Pakistan has faced several *N. fowleri* outbreaks in the past, causing serious public health issues. In several parts of Pakistan, the temperature can rise beyond 50°C,^4^ and the monsoon season, which is characterized by increased rainfall and stagnant water,^5^ also has an impact on the epidemiology of the disease there (39). A significant number of cases were reported in Karachi which has particularly scorching temperatures (40), most of them being reported between April and September when temperatures are peaking (41).

It is also noteworthy that individuals are more likely to engage in water-related activities such as swimming during the hot summer months, thereby increasing the likelihood of contracting *Naegleria*.

A study revealed that the water chlorination levels in Karachi's municipal water supply fell below the World Health Organization's recommended level of 0.5 ppm (41). Moreover, a significant number of individuals, particularly those from rural backgrounds, rely on unfiltered and non-chlorinated groundwater, which can act as a conducive breeding environment for *N. fowleri*. Pakistan is a predominantly Muslim country, and the use of groundwater and contaminated tap water for ritual ablution is a crucial factor contributing to the increase in *Naegleria* infections within the country.

## 7. Global geographic spread of infection

PAM is an uncommon but lethal disease that primarily affects young adults in wealthy nations but has also lately been documented in poorer nations, with a 95–99% fatality rate (2). After four patients died in Australia's Adelaide Children's Hospital in 1965, Fowler and Carter became the first to characterize PAM (19). An ameba invasion of their meninges, which caused significant damage and inflammation in the brain, was identified as the cause of death (42–44). PAM has since been recorded in other nations, with an estimated 400 victims globally. The total number of cases, however, is unknown and may be higher because of incorrect diagnoses or unreported cases (45–47). Except for Antarctica, 15 countries throughout the world have reported PAM cases (48, 49). These cases, which only number a few hundred, are primarily from Europe, Australia, the United States, and several Asian nations (13). Despite the stability of the reported infections in the United States each year (0–8), recent alterations in the epidemiology of PAM are quite concerning (50). In 2010, a PAM case from the northern state of Minnesota was reported for the first time. In 2011 and 2012, additional cases from Indiana, Minnesota, and Kansas were then reported, raising concerns about the infection's potential to spread across a wider geographic area due to the heat-loving, potentially climate-sensitive pathogen (51). In the United States, 142 PAM patients were recorded between 1937 and 2013. Approximately 260 cases have been reported worldwide between 1962 and 2014, according to a comprehensive study. Of these, 132 cases originated from the United States, 19 from Australia, 17 from Pakistan, 16 from the Czech Republic, 11 from India, 9 from Mexico, 9 from New Zealand, 7 from Venezuela, 5 each from Thailand and Belgium, 4 from Nigeria, 2 from the United Kingdom, and 1 case each from Namibia, Iran, Costa Rica, New Guinea, South Africa, and Madagascar. Out of the 260 documented cases (50, 52), just 11 individuals were said to have survived. The US Center for Disease Control and Prevention (CDC) reports that there were 138 PAM cases in the US between 1962 and 2015 (53). A recent rise in PAM instances has been observed in Asian nations.

## 8. *Naegleria fowleri* outbreak in Pakistan

**Figure 4:**
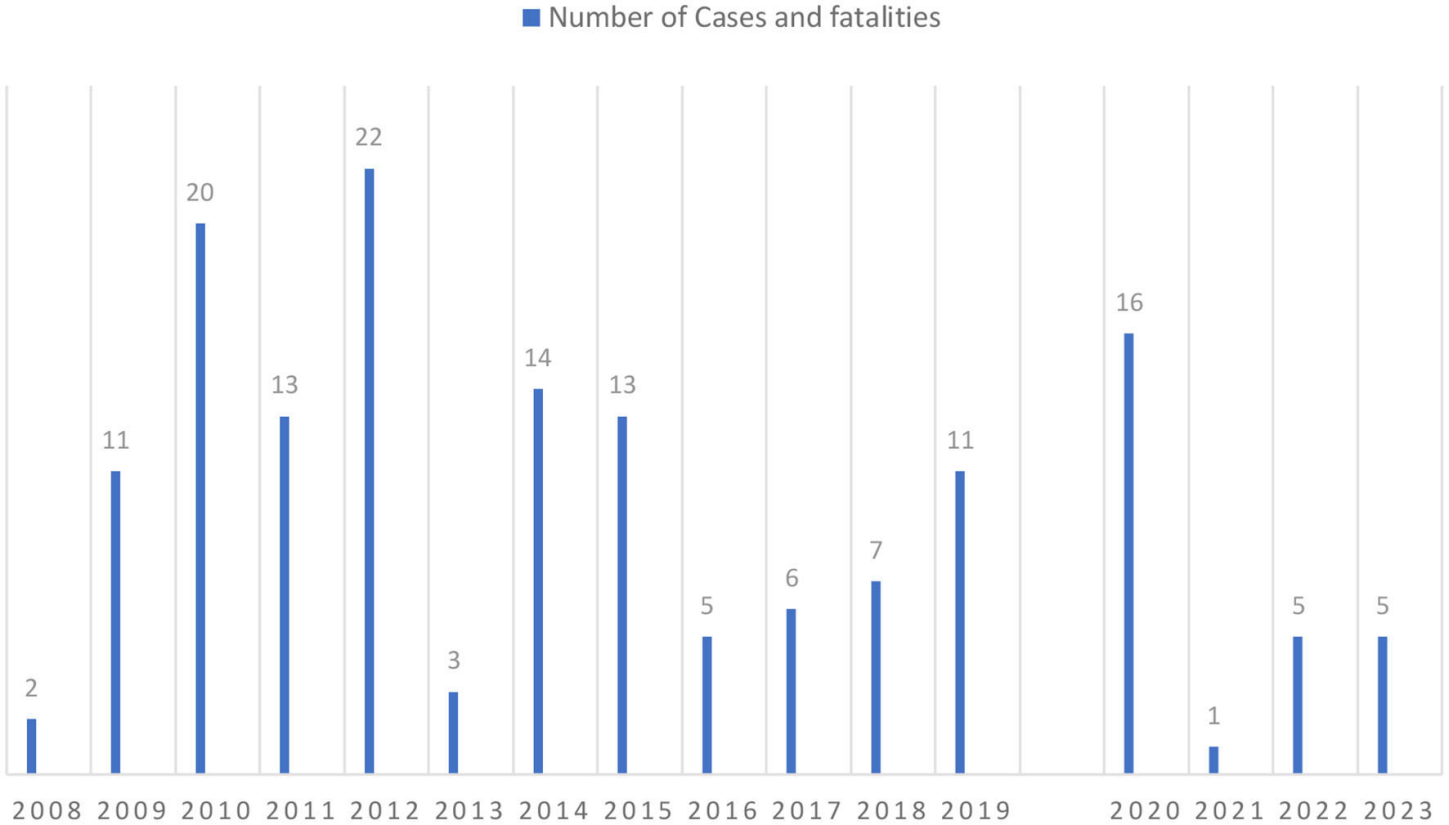
Reported cases of PAM in Pakistan from 2008 to 2023.

In Karachi, Pakistan's largest city, this ameba has been a growing concern (54). The Aga Khan University Hospital in Karachi, Pakistan, reported a noticeable death rate of about 20 deaths per year caused by PAM in Pakistan (2). As shown in Table 2 and Figure 4, from 2008 onwards *N. fowleri* has consistently been associated with a variable number of cases and fatalities.

**Table 2:** Reported cases and fatalities of primary amebic meningoencephalitis (PAM) in Pakistan (2008-2023) by year and gender.

**Year**	**Number of cases and fatalities**	**Age group-gender**	**References**
2008	2 cases	Age group: 30-y-old, 25-y-old Gender: all are males	(24)
2009	11 cases	Age group: from 16 to 64 y	(55)
2010	20 cases	Age group: not reportedGender: all are males	(56)
2011	13 cases	Age group: not reportedGender: all are males	(24)
2012	22 cases	Gender: all are males	(41)
2013	3 cases	Age group: from 14 to 40 y Gender: all are males	(41)
2014	14 cases	Age group: from 14 to 40 yGender: all are males	(41)
2015	13 cases	Age group: from 16 to 56 yGender:10 males, 3 females	(41)
2016	5 cases	Not reported	(57)
2017	6 fatalities	Not reported	(57)
2018	7 fatalities	Not reported	(58)
2019	11 fatalities	Age group: from 21 to 45 y Gender: 10 males	(58)
2020	16 cases	Not reported	(59)
2021	1 case	Age:19 y old	(60)
2022	5 cases	Age: 2 cases of 59 y old, 1 case of 38 y old, 1 case of 28 y old Gender: 2 males, rest not reported.	(54)
2023	5 cases	Age: 1 case of 21 y old, 1 case of 45 y old Gender: 1 female, 2 males, rest not reported.	(61)

Up to October of the year 2019, fifteen fatalities were documented (58, 62). 2020 had no confirmed deaths, although there is no assurance because of a lack of data collecting and unreported instances. In 2021, six fatalities were recorded (63). As of July 1, 2022, there have been four fatalities (64).

## 9. Factors contributing to the outbreak

The environment where *N. fowleri* infection is most likely to occur is characterized by warmer, polluted water. Infectious trophozoites can enter the body through the nose, cross the cribriform plate, and enter the human brain, where they can cause serious CNS damage, brain hemorrhage, and eventually death within 3–7 days. Moreover, this illness is made more deadly by delayed diagnosis and a lack of effective treatment options (2). Since the 1970s, researchers have known that *N. fowleri* and other free-living ameba multiply best between 30–42°C (65–67). When compared to cultures cultivated at the ideal 37°C, *N. fowleri* has also been proven to endure temperatures up to 45°C, however with reduced survival (66, 68). Environmental investigations have established that *Naegleria* can be found in thermal saline baths and naturally low-salinity water (68, 69). Lam et al. determined that after 48 h *N. fowleri* could survive in moderate salinity of up to 1.4%. This is roughly half as salty as ocean water and three to four times as salty as saltwater swimming pools (70).

## 10. Diagnostic modalities for *Naegleria fowleri* and primary amebic meningoencephalitis

It is difficult to treat *Naegleria* patients because of the rarity of the infection and the challenges in initial detection. Usually, the parasite is detected posthumously in the CSF samples of the victim using microscopy and advanced referral testing techniques. To form a definitive diagnosis, PAM can be confirmed in patients through investigations mentioned in Table 3 below.

**Table 3:** Detection methods for *Naegleria fowleri* in clinical and environmental samples.

**Detection method**	**Description**
Direct visualization	The motile ameba can be observed under a microscope in a fresh sample of cerebrospinal fluid (CSF).
Antigen detection	Specific antibodies can be used in conjunction with immunohistochemistry (IHC) or indirect immunofluorescence (IIF) to directly stain amebic antigens in tissue.
Polymerase chain reaction (PCR)	Specific molecular tools can amplify DNA from the ameba in CSF or tissue.
Ameba culture	The ameba can be grown into culture, increasing the likelihood of detecting it by direct visualization or PCR.
Environmental detection	Water samples can be collected, concentrated, and put into culture to grow and select for *N. fowleri*.

Typically, cultivation and confirmation of *N. fowleri* in the cerebrospinal fluid (CSF) are required for the laboratory diagnosis of PAM. A flagellation test (FT) is also frequently employed as an additional diagnostic method for *N. fowleri*. Due to the prevalence of some false negatives, FT must be followed by Enzyme-linked immunosorbent assay (ELISA) or another diagnostic technique [PCR, Restriction fragment length polymorphism (RFLP)] following both positive and negative results (71).

Diagnostic techniques based on ELISA typically only offer postmortem, retrospective, and late diagnoses. The genus *Naegleria*'s various restriction profiles serve as the foundation for the species-specific diagnostic technique known as RFLP. This method's advantage is its capacity to diagnose not only *N. fowleri* but also other *Naegleria* genus species. In the middle of the 1990s, a DNA probe-based detection approach was described (72). A useful alternative to microscopy and culture, molecular approaches are generally quite sensitive and may enable the discovery of microorganisms that are challenging to identify.

Most of these procedures have limitations, though, such as taking a long time (cultivation), being expensive (RFLP), only offering a late and retrospective diagnosis (ELISA), or yielding insufficient data (flagellation test). Many of these issues are resolved by PCR diagnostic techniques, which are helpful for the diagnosis of clinical and environmental specimens. One of the main benefits is that these PCR procedures take less time because it is possible to isolate DNA from the sample without prior culture. Additionally, PCR permits not only the detection of *N. fowleri* but also the distinction between other species of the *Naegleria* genus. Real-time PCR-based diagnostic techniques have the advantage of producing results quickly and allowing for real-time amplification process observation (73).

LAMP, or loop-mediated isothermal amplification, is a recent technique for amplifying DNA. LAMP is a gene amplification assay that is extremely specific, sensitive, quick, and repeatable. LAMP has several key advantages over other approaches, including ease of use, the ability to produce many precise amplification products at a consistent temperature without the need for expensive equipment, and the ability to visually assess the outcome of an amplification response. In clinical laboratories or field studies, such a test could be a helpful diagnostic tool, particularly in countries with limited resources. LAMP has been extensively utilized to find protozoan infections. Only one study proves that LAMP can be used to detect *Naegleria* in place of conventional PCR tests. This is because only a few of the putative DNA targets of the parasite have been investigated, probably because PAM is a rare disease (74).

Furthermore, patients with *N. fowleri* infections have been seen to have CSF pressures as high as 600 mm^3^ H_2_O. Due to a considerable rise in red blood cells, CSF analysis reveals a variety of color abnormalities, ranging from gray in the early stages of infection to red in the late stages. Additional increases are noted in trophozoites present in the CSF (using trichrome or Giemsa stain) and polymorphonuclear cell concentrations (as high as 26,000 mm^3^). The midbrain and subarachnoid space are two areas of the brain that Magnetic resonance imaging (MRI) frequently reveals to be abnormal (28, 29) in PAM.

## 11. Treatment options available

Since the discovery of primary amebic meningoencephalitis, numerous antifungal, antiprotozoal, antibacterial, and antipsychotic therapeutics have been tested against *N. fowleri*. Most of these medications were shown to be ineffective or hardly effective against *N. fowleri*, both *in vitro* and *in vivo*. Even though Amphotericin B (AmB) is still the drug of choice for treating primary amebic meningoencephalitis, using it is usually linked to renal damage, which manifests as azotemia and hypokalaemia. AmB frequently results in anemia, and many patients also have headaches, nausea, vomiting, chills, and fever. Additionally, not all AmB-treated patients have recovered from primary amebic meningoencephalitis (75).

Azithromycin (AZM) has demonstrated *in vivo* action against experimental toxoplasmosis as well as *in vitro* activity against *Acanthamoeba* spp. By attaching to the bacterial 50S ribosomal subunit and preventing the creation and translocation of peptide bonds, AZM prevents the production of proteins by bacteria. However, the mechanisms of action of AZM in *Acanthamoeba, Naegleria*, and *Toxoplasma* spp. have not been determined and require further research (75).

In combination with some of these other medications, the anti-leishmanial and breast cancer medication, miltefosine (MLT), has shown some promise against free-living amebae. MLT has demonstrated amebicidal activity against *Balamuthia, N. fowleri*, and *Acanthamoeba in vitro* and in mice models. It has been used to successfully treat patients with disseminated *Acanthamoeba* infection and *Balamuthia* infection. At high doses, patients taking MLT may experience nausea, vomiting, or diarrhea. It is somewhat nephrotoxic meaning that people with compromised kidney function may need to have their dosage modified and the risk for nephrotoxicity should be weighed against the risk for PAM-related mortality. It is important to note that there is still limited data on the dose of MLT that effectively treats amebic infection (76).

Auranofin, an anti-rheumatic medication, is amebicidal against *N. fowleri*. Auranofin treatment of *N. fowleri* cultures resulted in decreased ameba numbers, increased metabolic activity, and enhanced cell permeability at biologically relevant concentrations of 0.75–3.0 g/ml. These findings imply that the inclusion of auranofin in the treatment regimen for *N. fowleri*-infected individuals experiencing PAM may be beneficial (77, 78)

Staurosporine (STS), an indolocarbazole isolated from the bacteria *Streptomyces sanyensis*, has also demonstrated high activity against *N. fowleri* trophozoites *in vitro*. It has been suggested that its amebicidal activity occurs by inhibiting the ameba's protein kinase (PK) and inducing an apoptosis-like mechanism via the mitochondrial pathway, but more studies are required to confirm the mechanism of action (19).

Another drug with potential against *N. fowleri* trophozoites is pitavastatin. This medication has shown EC50 values against five different strains that range from 0.3 to 4 μM, demonstrating that it is more potent than miltefosine (EC50 values between 15 and 58 μM), but less potent than AmB (EC50 values between 0.06 and 0.2 μM). Pitavastatin decreases the development of the ameba *in vitro* by more than 60% as early as 10 h after exposure. Inhibition reaches 81% after 16 h and 96% after 24 h. Pitavastatin is an FDA-approved cholesterol-lowering drug, therefore its effects on various human cell lines have been well-studied (19, 79).

In a more recent investigation, different antibiotics and antifungals that could be used to treat PAM were evaluated using a high-throughput phenotypic screening method. The antifungal posaconazole (PCZ) stood out amongst these medications. This substance has an IC50 value of 0.24 μM and can stop an ameba's growth in about 12 h. Although no synergy was seen, PCZ also showed additive activity with AZM, AmB, and MLT. Additionally, sick mice who received 20 mg/kg of PCZ intravenously had a 33% survival rate; however, when PCZ and AZM were combined, the survival time was dramatically increased (80, 81).

Fluconazole, rifampin, and dexamethasone are some other drugs used in combination with amphotericin B. To kill the ameba inside the CNS, however, a minimum inhibitory concentration (MIC) of these medications must be provided, and they have demonstrated poor blood-brain barrier penetration (19).

The limited efficacy that many medications display because of their failure to successfully cross the BBB is one of the key issues associated with CNS infections. Sometimes, the solution to this issue is to deliver more of the medicine, however doing so may cause cell toxicity. Since they can increase a drug's effectiveness and enable a reduction in dosage, nanoparticles have drawn interest from the pharmaceutical sector. Studies have been conducted to understand the benefits of employing medications conjugated with nanoparticles to treat PAM because nanoparticle drug delivery methods have been shown to boost bioavailability, minimize cell toxicity, and are site-specific (19).

Recently, AmB and nystatin (NYS) coupled with silver nanoparticles (AgNp) have been shown to be more effective against *N. fowleri* than on their own. Apart from that gold nanoparticles (AuNp) are also being investigated as potential drug delivery techniques to treat PAM. In one study, AuNp was combined with curcumin to test its effectiveness against *N. fowleri* because the compound has anti-inflammatory characteristics and can reduce lipid peroxidation. At a concentration of 200 μM, curcumin exhibited a 66% amebicidal activity against *N. fowleri* because of concentration-dependent action. The bioavailability of curcumin was greatly increased by conjugation with AuNp, and this led to a 69% amoebicidal activity at a concentration of 10 μM. The human keratinized skin cells were not cytotoxically affected by curcumin conjugated with AuNp. Additionally, the secondary plant metabolite trans-cinnamic acid, which is derived from plants, has been conjugated with AuNp. Green nanoparticles created using green chemistry have also been investigated as potential safer and more environmentally friendly medicine delivery systems. Therefore, more recently, plant-derived polysaccharides such as gum tragacanth (Gt) and gum acacia (Ga) have been used to stabilize metal nanoparticles. Hesperidin (HDN) and Naringin (NRG), two flavonoids with antioxidant and anti-inflammatory properties that may help lessen the immunopathogenic process released during PAM, were conjugated to the green nanoparticles. In comparison to the nanoparticles alone, both Ga-AgNPs-HDN and Gt-AuNPs-NRG have shown substantial amebic activity against *N. fowleri* (19).

## 12. Impact on communities

Both communicable and non-communicable disorders are already overburdening Pakistan's healthcare system. Pakistan is one of the few nations where poliovirus is still prevalent and at this point, if a *Naegleria* epidemic occurs, it would further exacerbate already existing gaps in the healthcare system (54).

Pakistan has experienced massive outbreaks of diseases like diarrhea, dengue, malaria, polio, and COVID-19 amid record floods that have harmed over 900 medical facilities and affected 33 million people. The nation has the second-highest global hepatitis C burden.^6^ Following the floods, Pakistan has seen an increase in both malaria and dengue cases and 78% of all confirmed malaria cases in 2022 occurred in Sindh and Balochistan (82). Flood water stands still and can be a breeding ground for *Naegleria*. The hot temperature of Punjab and Sindh province of Pakistan further boost survival conditions for *N. fowleri*. The optimal temperature range for *N. fowleri* growth is up to 115°F (46°C) and it may endure greater temperatures for brief periods even though it may not be able to develop as effectively. The trophozoites and cysts may endure 122–149°F (50–65°C) for minutes to hours, with the cysts being more resilient (35). The community can face harsh consequences due to an *N. fowleri* outbreak. This is keeping in mind that the public and economy still haven't been able to move on from the damage imposed by previous outbreaks like malaria and dengue which were also due to massive floods in Pakistan.

The cases of *N. fowleri* infections that have occurred in Pakistan during the past 8 years have not been completely described by any study. Most of the data for the nation is either missing or not reported. The healthcare system in Pakistan is already overstrained. The system has a lack of employees and is underfunded. Since PAM progresses quickly, most patients have clinically deteriorated by the time the diagnosis is made. Due to the disease's rarity, the CDC reports that autopsies performed after patient deaths confirm the diagnosis in 75% of instances. After the development of clinical symptoms, the patient only has a very small window of time for therapy to be effective. Given the exceedingly high death rates associated with PAM (almost 100%), it is likely that many individuals seek medical care at a point where current treatments are useless. The available information summarized in Section 8, Table 2, and Figure 4 indicates that there have been more deaths in recent years, thus reiterating the need for action to combat the increasing trend.

## 13. Overcoming the crisis

No significant measures are being taken by the Government of Pakistan to counter the *N. fowleri* outbreak because of the rarity of the disease and absence of definitive treatment. But during 1970s and 1980s cases of *Naegleria* associated PAM came up in Australia (83). There, drinking water was distributed by overland pipes for hundreds of kilometers before it arrived at household faucets with no discernible residual disinfection. Implementing an ameba monitoring scheme and raising disinfection residual levels was the successful Australian reaction to these PAM incidents (19); nevertheless, certain systems necessitated switching to chlorination to maintain disinfectant residual levels over extended distances (89). The health authorities of Pakistan should adopt the methods used by the Australians with the hope of ensuring the safety of the civilians and countering this outbreak. The concerned authorities could also set up an ameba monitoring system and an efficient chlorination system. The chlorination facility is available in Pakistan but no check and balance is implemented for its proper working. Moreover, for the first time, a PAM-associated death was linked to a U.S. treated drinking water system that had culturable *N. fowleri* in it (84). Previously, in 2011, one neti pot PAM incident occurred in the same US area of St. Bernard Parish, Louisiana and this discovery sparked an environmental assessment of the household and parish water distribution system, and the results finally revealed the presence of *N. fowleri* along with numerous undetected disinfectant residual areas (84).

Water utility and health officials decided that a temporary chlorine conversion was necessary to inactivate *N. fowleri* and reduce biofilm in the distribution systems because the St. Bernard and DeSoto Parish water utilities used chlorination to maintain a disinfectant residual in their systems. According to health officials, *N. fowleri* should not grow if a free chlorine level of 2.0 mg/L is maintained in all storage tanks for at least 60 days and a free chlorine level of 2.0 mg/L is maintained at all points in the distribution system. Each water system turned off the ammonia feed and boosted the free chlorine feed to start the chlorine conversion process after warning the public about the upcoming conversion of chlorine. Disinfectant residuals in the St. Bernard and DeSoto distribution systems met or exceeded the 1 mg/L free chlorine objective following the chlorine conversion (85). Since *N. fowleri* is vulnerable to chlorine inactivation, no *N. fowleri* was found in either system after the chlorine conversion. Conditions in the St. Bernard distribution system favored *N. fowleri* growth and persistence. The distribution system's water temperatures rose, thus allowing *N. fowleri* to flourish (86). It was challenging to keep the disinfectant residual consistent throughout the distribution system, as seen by a decline in average concentration over time. Over the course of the year, Heterotrophic Plate Count (HPC) concentrations also increased. HPC is widely regarded as a good measure of biofilm formation and is an indicator of bacterial regeneration inside a distribution system (87). Because *N. fowleri* easily forms biofilms and survives there, more residual or contact time with the disinfectant is needed to sufficiently inactivate it (85). Therefore, a rise in HPC over time suggests that *N. fowleri* may be present and that the distribution system's circumstances are conducive to biofilm formation and persistence. According to a 1984 study, the abundance of *N. fowleri* in drinking water distribution systems was inversely correlated with chlorine residual and positively correlated with water temperature and colony counts at 35°C (like HPC) (88). *N. fowleri* was both eliminated and prevented from recolonizing in bulk water and biofilm at a constant free chlorine concentration of >1 mg/L (85). The discovery of non-viable *N. fowleri* in the St. Bernard system in September suggests that the circumstances in the distribution system were suitable for containing *N. fowleri*. To find out what other elements contribute to the discovery of *N. fowleri* in drinking water distribution systems, further research is required. If such a chlorination system is recruited by the government of Pakistan then combating *N. fowleri* outbreak will be quite easy. Managers of water distribution systems should be knowledgeable about the ecology of their systems, be aware of any changes in water quality brought on by rising water temperatures, and seek to minimize any locations where biofilm formation may be an issue and have an impact on water quality (89). There aren't any biofilm monitors (such as) for water in pipelines in Pakistan but to avoid a dangerous *N. fowleri* outbreak such monitoring systems should be installed. Also, strict measures to chlorinate tap water should be adopted to eliminate any parasites existing in this supply.

The public should take extra precautions when using tap water for nose or sinus cleaning because there is no guarantee that even the most careful drinking water system can entirely eradicate *N. fowleri*. At the point of use, water used for nasal, or sinus rinses should receive additional treatment. This can include boiling the water for 1 min and letting it cool before using, filtering the water through a device with an absolute pore size of one micron or less, disinfecting the water with chlorine bleach, or using distilled or sterile water (89). Furthermore, if swimming in water bodies can at no cost be avoided then one should put nose plugs in use to prevent water entering the nose. In addition, whilst swimming one's head should be held up above the surface of water. It would be better to ensure that chlorine levels of the water body are appropriately high to keep the parasite out of it (20).

## 14. Lessons learned and recommendations

To build resilience for future outbreaks and to promote early detection of *N. fowleri* in Pakistan, both of which mitigate the potential dangers associated with this lethal ameba, it is important that we first evaluate the gaps in preparedness and response mechanisms to *N. fowleri* in Pakistan and then work toward filling those gaps.

Pakistan is a developing country with a high percentage of people living under the poverty line (90). It has a literacy rate of only 62.3% which indicates that at present, ~60 million people are illiterate in the country.^7^ Public awareness and education are two major areas that require attention. The existence and consequences of *N. fowleri* are still largely unknown among the general public in Pakistan.

The deficiencies in our healthcare system also need to be dealt with. These include rural-urban disparity in healthcare provision, lack of essential diagnostic and therapeutic facilities, and a low doctor-population, nurse-population, and hospital beds to population ratio in the country (10).

Third, as mentioned before, poorly maintained and chlorinated water supply systems, swimming pools, and recreational water bodies are all possible breeding grounds for *N. fowleri* and serve as sources of infection. This is a major challenge in Pakistan where water surveillance and water standards are far from satisfactory (9).

To bridge these gaps and to safeguard public health in Pakistan it should be ensured that information on symptoms, preventive measures like the use of nose clips and water chlorination, and the value of early diagnosis and treatment, which have been shown to increase survival rates (91) is communicated widely among the people. This can be done by way of public health advertisement campaigns on social media, TV, radio, and newspapers in addition to conducting informative sessions about the disease.

The current health expenditure in Pakistan is only 2.95% of the GDP^8^ and a greater budget needs to be allocated to healthcare to deal with the various problems associated with it. The formation of well-equipped healthcare centers and laboratories along with the training of health workers to promptly identify and formulate effective treatment regimens for *N. fowleri* patients is of vital importance. It must also be made certain that all patients, including those living in far-off rural areas, have access to appropriate medical services to help tackle the infection.

Besides that, laws should be set in place tighten water surveillance and improve water quality in line with WHO standards,^9^ and greater investment in water infrastructure also needs to be made.

Finally, and most importantly, cooperation and a joint, collaborative effort between the various interested parties including the public, governmental organizations, healthcare providers, and water supply authorities is imperative and will help to ensure an effective and systemized response even if an *N. fowleri* epidemic arises in the future.

## 15. Role of research and public health organizations in *Naegleria* prevention and control

Research institutions play a crucial role in providing up-to-date information necessary for a better understanding of *N. fowleri*. This knowledge contributes to the development of effective preventive measures, targeted therapies, and advancements in disease detection and diagnosis. Through studies on the prevalence and distribution of disease, research institutions can also help identify disease patterns and high-risk groups. Additionally, they can investigate water quality and contamination to identify potential sources of infection. Furthermore, research allows for the evaluation of the effectiveness of preventive measures and treatment options.

Consequently, establishing a strong link between research institutions and public health organizations is vital in creating comprehensive strategies that encompass prevention, monitoring, and rapid intervention to safeguard the public from the threat of *N. fowleri*. Collaboration and teamwork between these entities facilitate the pooling of resources and data. This collaborative effort leads to the formulation of policies and treatments that are firmly grounded in scientific evidence, thereby improving the overall health of the general population. Moreover, this collaborative approach aims to reduce or eliminate inequalities in healthcare, ensuring that all individuals have access to appropriate care and resources (92).

## 16. Conclusion

In conclusion, the *N. fowleri* crisis in Pakistan has exposed an alarming situation that requires urgent action and recovery initiatives. The devastating impact of this brain-eating ameba on public health necessitates a comprehensive and well-executed action plan. Despite this, through collective efforts and an unrelenting dedication to public health, there is hope that the country can overcome this calamity. The outbreaks of *N. fowleri* in Pakistan have compounded existing healthcare burdens and environmental issues, making it imperative for the government to take proactive measures. Implementing an ameba monitoring system and efficient water chlorination are crucial steps to prevent further spread of the ameba.

Public awareness campaigns and education are vital to inform the population about preventive measures and the significance of early diagnosis and treatment. Collaboration between research institutions and public health organizations is crucial in formulating evidence-based policies and treatments, ensuring the safety and wellbeing of the people.

## Author contributions

AN: Conceptualization, Writing—original draft, Writing—review and editing. IM: Writing—review and editing. EA: Writing—review and editing. FS: Writing—review and editing.

## Footnotes

^1^*Reemergence of Deadly Brain-Eating Amoeba Sets Off Alarms*. Available from: https://tribune.com.pk/story/2425212/reemergence-of-deadly-brain-eating-amoeba-sets-off-alarms (accessed July 11, 2023).

^2^*Pakistan Man Dies From ‘Brain-Eating Amoeba' After Going for a Swim*. Available from: https://www.telegraph.co.uk/global-health/science-and-disease/pakistan-amoeba-man-dies-naegleria-fowleri/ (accessed July 11, 2023).

^3^BioRender. Available online at: https://app.biorender.com/user/signin (accessed July 14, 2023).

^4^*Pakistan Records Hottest Day Ever in Turbat Area of Balochistan. Al Arabiya English*. Available online at: https://english.alarabiya.net/News/world/2017/05/29/Pakistan-records-hottest-day-ever-in-Turbat-area-of-Balochistan (accessed September 6, 2023).

^5^*Pakistan - Climatology. Climate Change Knowledge Portal*. Available from: https://climateknowledgeportal.worldbank.org/country/pakistan/climate-data-historical (accessed September 6, 2023).

^6^*Pakistan Faces Widespread Outbreaks of Infectious Diseases Amid Unprecedented Floods*. Available online at: https://www.news-medical.net/news/20220909/Pakistan-faces-widespread-outbreaks-of-infectious-diseases-amid-unprecedented-floods.aspx (accessed July 14, 2023).

^7^*Ministry of Federal Education and Professional Training*. Available online at: https://mofept.gov.pk/ProjectDetail/NjQ4ZTg2NjItOWM2NC00Y2IxLTkzMDgtMjU2OTFhMjA4NzNh (accessed July 13, 2023).

^8^*Current health expenditure (% of GDP) - Pakistan*. Data. Available online at: https://data.worldbank.org/indicator/SH.XPD.CHEX.GD.ZS?locations=PK (accessed July 13, 2023).

^9^*Water Sanitation and Health*. Available online at: https://www.who.int/teams/environment-climate-change-and-health/water-sanitation-and-health/water-safety-and-quality (accessed July 13, 2023).
